# A climate vulnerability assessment of the fish community in the Western Baltic Sea

**DOI:** 10.1038/s41598-024-67029-2

**Published:** 2024-07-13

**Authors:** Dorothee Moll, Harald Asmus, Alexandra Blöcker, Uwe Böttcher, Jan Conradt, Leonie Färber, Nicole Funk, Steffen Funk, Helene Gutte, Hans-Harald Hinrichsen, Paul Kotterba, Uwe Krumme, Frane Madiraca, H. E. Markus Meier, Steffi Meyer, Timo Moritz, Saskia A. Otto, Guilherme Pinto, Patrick Polte, Marie-Catherine Riekhof, Victoria Sarrazin, Heike Schwermer, Marco Scotti, Rudi Voss, Helmut Winkler, Christian Möllmann

**Affiliations:** 1 Thuenen Institute of Baltic Sea Fisheries, Rostock, Germany; 2 Alfred-Wegener Institute for Polar and Marine Research, Wadden Sea Station Sylt, List, Germany; 3 Institute of Marine Ecosystem and Fishery Science, Center for Earth System Research and Sustainability (CEN), Hamburg University, Hamburg, Germany; 4 Marine Ecology Research Division, GEOMAR Helmholtz Centre for Ocean Research Kiel, Kiel, Germany; 5 Department of Physical Oceanography and Instrumentation, Leibniz Institute for Baltic Sea Research Warnemünde, Rostock, Germany; 6 BioConsult GmbH & Co. KG, Bremen, Germany; 7 Stiftung Deutsches Meeresmuseum – Museum für Meereskunde und Fischerei, Deutsches Meeresmuseum, Stralsund, Germany; 8 German Centre for Integrative Biodiversity Research (iDiv), Halle-Jena-Leipzig, Leipzig, Germany; 9 Center for Ocean and Society (CeOS), Christian-Albrechts-University Kiel, Kiel, Germany; 10 Leibniz Institute for Biodiversity Change Analysis (LIB), Museum of Nature – Zoology, Hamburg, Germany; 11 Institute of Biosciences and Bioresources, National Research Council of Italy, Firenze, Italy; 12 Department of Zoology, University of Rostock, Rostock, Germany

**Keywords:** Climate change, Climate vulnerability assessment, Fish community, Trait-based sensitivity, Western Baltic Sea, Ecology, Biodiversity, Biooceanography, Climate-change ecology, Community ecology, Conservation biology, Ecosystem ecology

## Abstract

Marine fisheries are increasingly impacted by climate change, affecting species distribution and productivity, and necessitating urgent adaptation efforts. Climate vulnerability assessments (CVA), integrating expert knowledge, are vital for identifying species that could thrive or suffer under changing environmental conditions. This study presents a first CVA for the Western Baltic Sea's fish community, a crucial fishing area for Denmark and Germany. Characterized by a unique mix of marine, brackish, and freshwater species, this coastal ecosystem faces significant changes due to the combined effects of overfishing, eutrophication and climate change. Our CVA involved a qualitative expert scoring of 22 fish species, assessing their sensitivity and exposure to climate change. Our study revealed a dichotomy in climate change vulnerability within the fish community of the Western Baltic Sea because traditional fishing targets cod and herring as well as other species with complex life histories are considered to face increased risks, whereas invasive or better adaptable species might thrive under changing conditions. Our findings hence demonstrate the complex interplay between life-history traits and climate change vulnerability in marine fish communities. Eventually, our study provides critical knowledge for the urgent development of tailored adaptation efforts addressing existing but especially future effects of climate change on fish and fisheries in the Western Baltic Sea, to navigate this endangered fisheries systems into a sustainable future.

## Introduction

Marine fisheries are vulnerable to climate change because of its impacts on the distribution and productivity of living marine resources^1^. As a consequence, adaptation efforts addressing existing but especially future effects of climate change are crucially required to navigate fisheries systems into a sustainable future^2^. Climate vulnerability assessments (CVA) based on expert knowledge contribute to adaptation planning because they may identify species potentially flourishing or suffering under future novel environmental conditions. CVAs are related to climate risk assessments that evaluate the potential impact of one or multiple hazards on species, habitats or ecosystems, either with a qualitative (based on expert opinion), semi-quantitative (based on data and expert opinion) or quantitative approaches (based on data only)^3^. In case of assessing the potential climate impact on entire fish communities where limited quantitative data is often available for a large number of local species, CVAs have proven efficient and been applied to many areas of the world ocean^4–9^. In CVAs vulnerability is usually assessed as the combination of trait-based sensitivity of individual species and their exposure to climate change due to spatial overlap with a hazard such as warming^9^.

Here we report on a first CVA for the fish community of the Western Baltic Sea, an important fishing ground for fisheries mainly in Denmark and Germany. The Western Baltic Sea is a transition area between the more saline areas bordering the North Sea (i.e. Skagerrak and Kattegat) and the low saline Baltic proper. Similar to most areas of the Baltic Sea, the Western Baltic Sea experienced significant climate-related warming that is expected to continue in the future^10,11^. Along with the warming more frequent and longer marine heatwaves are reported that may play a role in the appearance of local hypoxic areas^12–14^. Due to its dynamic physical oceanography^15^, the fish community of the Western Baltic Sea contains a peculiar mix of marine, brackish and freshwater species^16^. Traditionally stocks of cod (*Gadus morhua*) and herring (*Clupea harengus*) supported profitable local fisheries that provided income and cultural identity for coastal communities^17^. Recently, both stocks collapsed due to the combined effects of overfishing and climate change^18,19^ which caused an ongoing demise of local fisheries with important implications for local communities^17,20^. However, how future climate change will likely affect the entire fish community of the Western Baltic Sea is largely unknown. Consequently, knowledge is lacking about which species are critically endangered or may even be profitable resources under future novel environmental conditions. Lacking knowledge on the vulnerability of the Western Baltic fish community presently hampers efforts to transition the remaining fisheries towards a more sustainable future.

Our CVA involved a qualitative expert scoring of 22 fish species, assessing their sensitivity and exposure to climate change. Our study revealed a dichotomy in climate change vulnerability within the fish community of the Western Baltic Sea because traditional fishing targets and species with complex life histories are considered to face increased risks, whereas invasive or better adaptable species might thrive under changing conditions. Our findings hence demonstrate the complex interplay between life-history traits and climate change vulnerability in marine fish communities. Eventually, our study provides critical knowledge for the urgent development of tailored adaptation efforts addressing existing but especially future effects of climate change on fish and fisheries in the Western Baltic Sea, to navigate this endangered coastal fisheries systems into a sustainable future.

## Materials and methods

### Approach

**Figure 1:**
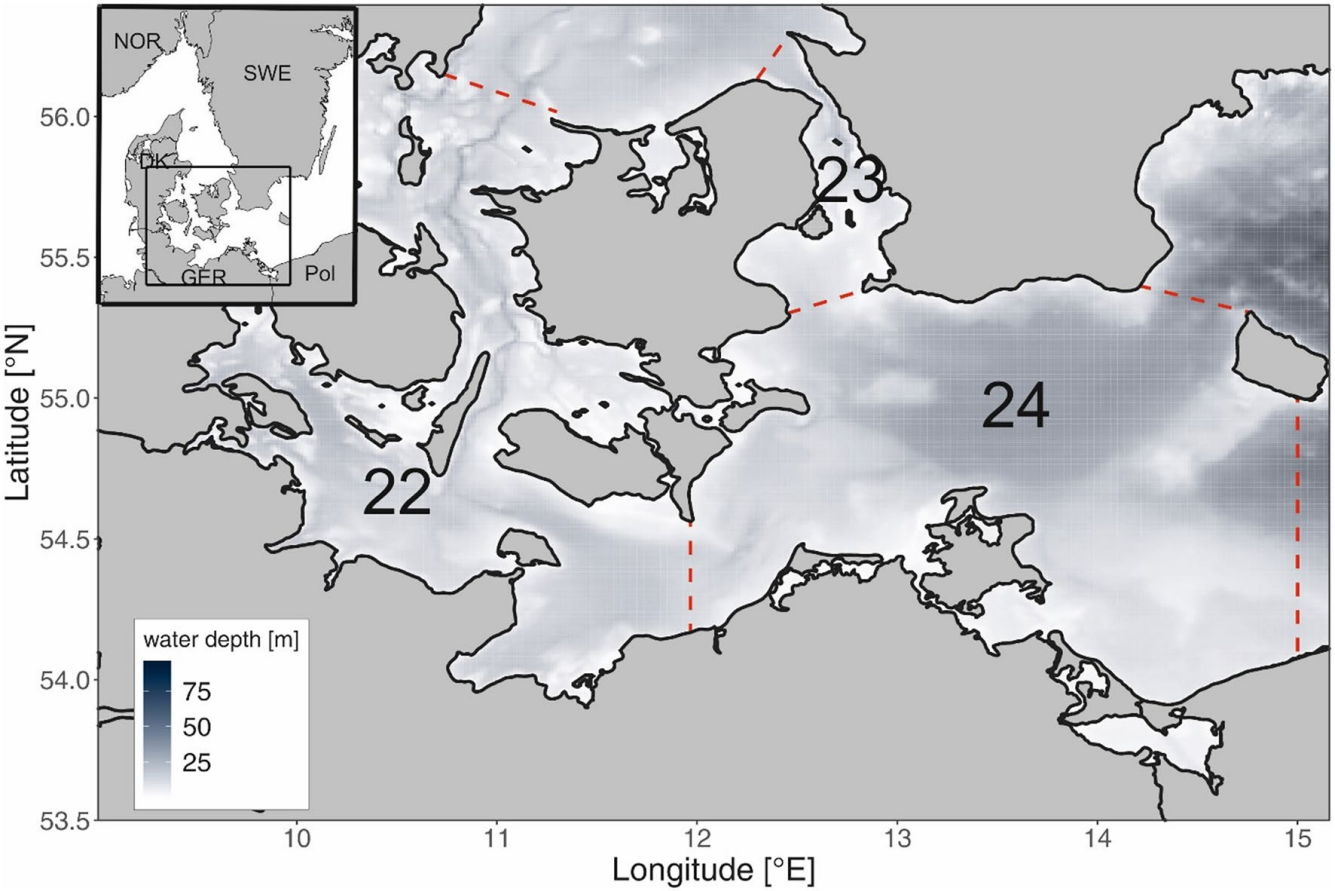
Study area. Map of the Western Baltic Sea with International Council for the Exploration of the Sea (ICES) management units (Sub-divisions: 22—Belt Sea, 23—Sound and 24—Arkona Sea).

We conducted a climate vulnerability assessment (CVA) for the fish community in the Western Baltic Sea, including the Belt Sea, the Sound and the Arkona Sea, covering the *International Council for the Exploration of the Sea* (www.ices.dk) sub-divisions 22–24 (Fig. 1). The Western Baltic Sea is a mesohaline transition zone (salinities of ~ 20 PSU in the Belt Sea and ~ 7–10 PSU in the Arkona Sea/Pomeranian Bight) between the species-rich marine waters of the Kattegat-Skagerrak and the rather species-poor brackish Baltic Proper^16^. We largely followed the CVA approach for fish communities developed for other areas that combines expert scorings of trait-based sensitivity of species with exposure scorings to locally important climate factors^6,9^. Similarly to these studies we considered adaptive capacity of the fish species to be covered by sensitivity attributes^9^.

Expert ratings of sensitivity were supported by detailed species profiles (see below—*Sensitivity*) that were prepared based on a comprehensive literature review. Species profiles were drafted by scientists within the *balt_ADAPT* project (“Adaptation of the Western Baltic Coastal Fishery to Climate Change”), who also conducted a preliminary test of the sensitivity scoring exercise. Afterwards species profiles were reviewed in a targeted workshop with additional experts on Baltic fish ecology external to the *balt_ADAPT* project. The final sensitivity scoring was developed by a combined group of *balt_ADAPT* and external scientists. Exposure scoring was conducted during two targeted project workshops by a sub-group within *balt_ADAPT* and prepared by analyses of available projections of physical-oceanographic variables^21^ and fish species distribution maps. Scoring exercises were conducted using online questionnaires prepared on the “Survey Solutions” online platform (https://mysurvey.solutions/en/). Background material on the questionnaire is available at https://github.com/GuiSPinto/cva_baltADAPT.

### Sensitivity

#### Species list and profiles

As a first step of our study we selected fish species to be included in the CVA based on monitoring data on the local fish communities from the Baltic International Trawl Survey (BITS), accessible through the DATRAS webportal (https://datras.ices.dk). Based on species distribution maps (see below—*Exposure*) we selected species that persistently occur in the Western Baltic Sea. We additionally included fish species with lacking population data (mostly coastal fish species not covered by BITS) when local experts confirmed their occurrence and/or habitat use for distinct life stages (early life stage and/or adult stage) within our study area. Our species list contained 22 commercially-exploited marine, but also non-exploited marine and freshwater/brackish fish species (Table 1).

**Table 1:** Fish species included in the climate vulnerability assessment for the Western Baltic Sea.

Picture	Scientific name	Common name	Ecotype
	*Gadus morhua*	Atlantic **cod**	Marine
	*Clupea harengus*	Atlantic **herring**	Marine
	*Sprattus sprattus*	European **spra**t	Marine
	*Merlangius merlangus*	**Whiting**	Marine
	*Scophthalmus maximus*	**Turbot**	Marine
	*Scophthalmus rhombus*	**Brill**	Marine
	*Limanda limanda*	Common **dab**	Marine
	*Platichthys flesus*	European **flounder**	Marine
	*Pleuronectes platessa*	European **plaice**	Marine
	*Solea solea*	Common **sole**	Marine
	*Scomber scombrus*	Atlantic **mackerel**	Marine
	*Chelon labrosus*	Thicklip grey **mullet**	Marine
	*Belone belone*	**Garfish**	Marine
	*Cyclopterus lumpus*	**Lumpsucker**	Marine
	*Zoarces viviparus*	**Eelpout**	Marine
	*Salmo salar*	Atlantic **salmon**	Marine-freshwater (anadromous)
	*Salmo trutta*	Sea **trout**	marine-freshwater (anadromous)
	*Anguilla anguilla*	European **eel**	Freshwater-marine (catadromous)
	*Perca fluviatilis*	European **perch**	Freshwater-brackish
	*Sander lucioperca*	**Pikeperch**	Freshwater-brackish
	*Esox lucius*	Northern **pike**	Freshwater-brackish
	*Neogobius melanostomus*	Round **goby**	Freshwater-brackish

Pictures are from https://fish-commercial-names.ec.europa.eu/fish-names/; bold parts of the common names are used for simplicity in the further course of the paper.

As a basis for the expert-based sensitivity scoring we developed profiles for each species based on an extensive literature research. Profiles contain comprehensive information with regard to 12 selected sensitivity attributes (Table 2). If available, we added distribution ranges within the study area as well as information on stock size, recruitment and growth to provide further background during the scoring process. We initially focused our literature research on local information for each fish species. However, in case regional information on certain biological traits of a species were lacking, we added information from adjacent and other regions of the world ocean. Fish profiles are available at https://github.com/GuiSPinto/cva_baltADAPT.

**Table 2:** List of sensitivity attributes and corresponding descriptions as baseline for the scoring process. Short versions of the attribute names in brackets as used in Fig. 5.

Biological attributes	Description	Low sensitivity	High sensitivity
Habitat specificity [habitat]	Relative dependence of a population to a habitat and the abundance of key habitats	Population is a habitat generalist and utilizes very common habitats during the entire ontogeny	Population is a specialist on an abundant habitat
Prey specificity [prey]	Population is a prey generalist or specialist	Population eats a large variety of prey (opportunistic feeder)	Population is partial to a single prey type (prey specialist)
Adult mobility [mobility]	Ability of the population to move if their current location becomes unsuitable (homing behavior excluded)	Highly mobile and non-site dependent adults	Site dependent adults with limited mobility
Dispersal of early life stages [dispersal]	Ability of the population to colonize new habitats	High egg and larvae dispersal	Low egg and larval dispersal
Early life history survival and settlement requirements [ELH]	Relative importance of early life history requirements for the population	Eggs and larvae have minimal requirements	Eggs and larvae have some specific requirements
Complexity in reproductive strategy [complexity]	Sensitivity of reproductive strategy to climate change. Reproductive characteristics are (e.g.): Diadromous migration, parental care/guarding nest behavior, homing behavior)	Simple reproductive strategy (no more than one characteristic)	Complex reproductive strategy (three characteristics)
Population spawning cycle [spawning]	Spawning strategies are sensitive to climatic changes (focus on the dominant population within the system)	Consistent throughout the year without a defined spawning season	One spawning event per year within a confined time frame
Sensitivity to temperature [temperature]	Known temperature of occurrence or the distribution of the species as a proxy for sensitivity to temperature	Wide temperature range throughout ontogeny and adapted to warmer water temperatures/no impact on spawning phenology	Somewhat limited temperature range throughout ontogeny and adapted to colder water temperatures/moderate/high impact on spawning phenology
Sensitivity to salinity [salinity]	Known salinity tolerance or the distribution of the species as a proxy for sensitivity to salinity	Euryhaline/life stages occurring in the Baltic Sea are euryhaline	Limited salinity range/life stages occurring in the Baltic Sea show a limited salinity range
Sensitivity to ocean acidification [acidification]	Sensitivity or tolerance against decreased pH (relationship to "sensitive taxa")	Population either does not rely on pH sensitive taxa (for food), or is expected to show no effect/impact to ocean acidification	Population is reliant on sensitive taxa and/or certain life stage is negatively affect by a decrease in pH
Population growth rate [growth]	Productivity of the population, using distinct parameters (max. growth rate, von Bertalanffy K, age at maturity, max. age, natural mortality M, measured max. length)	Population growth rate is high, high productivity	Population growth rate is low, low productivity, affected to any environmental changes
Additional stressors [other]	Other factors that could limit population responses to climate change (other than fishing pressure)	Population is experiencing limited stress (no/ no more than one known stressor, i.e., sensitivity to low oxygen, predation pressure, cannibalism, pollutants, anthropogenic stressors (eutrophication, habitat modification))	Population is experiencing moderate/high stress (no more than tow to three known stressors, i.e., sensitivity to low oxygen, predation pressure, cannibalism, pollutants, anthropogenic stressors (eutrophication, habitat modification))

#### Attributes and scoring categories

Sensitivity attributes are biological traits/life history characteristics that show the capacity of a species to respond to a changing environment^9^. We included a set of 12 sensitivity attributes referring to specific environmental requirements during fish life history and modified these to fit within the scope of the Western Baltic Sea ecosystem (Table 2). Furthermore, we developed descriptions for the four different scoring categories (bins), i.e. low, moderate, high and very high, representing the different degrees of sensitivity for each attribute (see below—*Data analysis*).

### Exposure

#### Climate exposure maps

We initially aimed at exploring the exposure of fish species to climate-induced changes in temperature, salinity and oxygen, because these three oceanographic factors are known to strongly influence Baltic Sea fish communities^16,25^. We hence explored oceanographic projections that are based on four regional climate models coupled to a regional three-dimensional ocean circulation model^21,26^. The horizontal and vertical resolutions of the ocean model are 3.7 km and 3 m, respectively. Initial explorative analyses did not reveal clear future climate-related trends in salinity and oxygen in the Western Baltic Sea, which confirmed earlier analyses of projection outputs revealing difficulties in modelling salinity^21^, and the dominating dependence of oxygen conditions on natural variability and eutrophication abatement strategies^26,27^. We hence focused our CVA on future climate-induced temperature changes (surface and bottom; °C).

**Figure 2:**
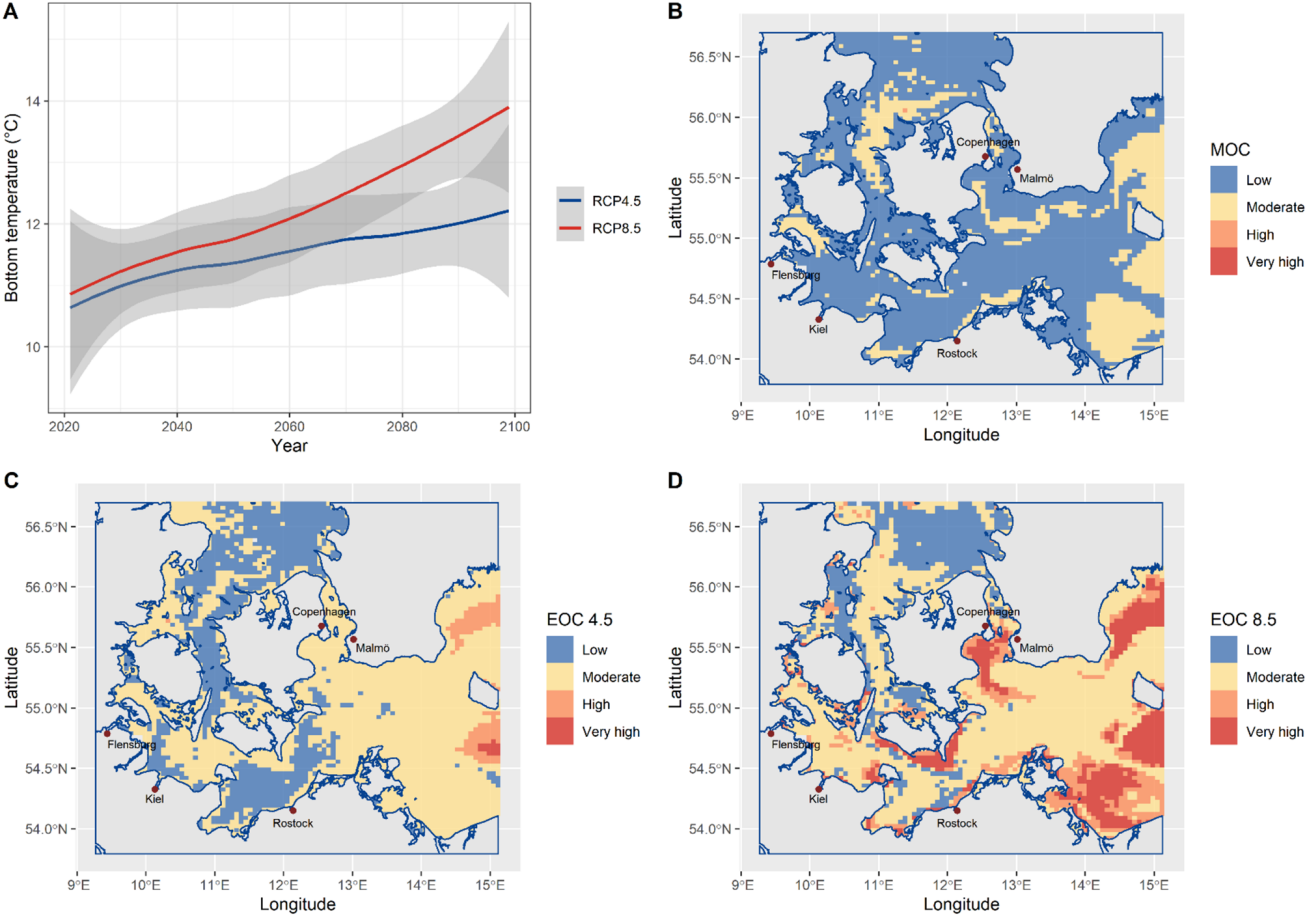
Future temperature changes in the Western Baltic Sea. Example maps illustrating future bottom summer temperature changes used to assess the exposure of the fish community. (**A**) Temperature projections for mean bottom temperature under emission scenarios RCP 4.5 and RCP 8.5. (**B**) Map of Z-scores for the “mid of century” (MOC, 2040–2049) scenario. (**C**) Map of Z-scores for the “end of century” (EOC, 2080–2089) scenario under RCP 4.5. (**D**) Map of Z-scores for the “end of century” (EOC, 2080–2089) scenario under RCP 8.5. Z-scores in (**B**–**D**) are categorized into *Low*, *Moderate*, *High* and *Very High*.

Seasonal and annual exposure maps were developed based on temperature projections according to Representative Concentration Pathways (RCP) scenarios RCP 4.5 and RCP 8.5^28^. We explored differences in mean bottom and surface temperatures for a “mid of century” (MOC, 2040–2049) and an “end of century” (EOC, 2080–2089) period in relation to a reference period (2010–2019). For the MOC analysis, we used RCP 4.5. only, since temperature trajectories of both RCPs only diverge later in the century^21^. We used Z-scores to measure the amount of future change relative to the reference period^6,9^:

1 $$\left|Z\right|= \left|\frac{{\overline{X} }_{f}- {\overline{X} }_{r}}{{\sigma }_{r}}\right|$$

where ${\overline{X} }_{f}$ is the mean of the future period, ${\overline{X} }_{r}$ is the mean of the reference period, and ${\sigma }_{r}$ is the standard deviation of the reference period. Similar to Spencer et al.^6^, Z-scores were classified into four exposure bin categories: low ($\left|Z\right|$ ≤ 0.5), moderate (0.5 < $\left|Z\right|$  ≤ 1.5), high (1.5 < $\left|Z\right|$  ≤ 2.0), and very high ($\left|Z\right|$ > 2.0). The direction of the changes in temperature was not considered since we only aimed at analyzing the amount of change. We mapped the data for all RCP and MOC/EOC combinations in a quartile fashion with mean layers^6^. In total 6 maps of annual means (Fig. 2) and 24 quarterly maps (indicating seasonal changes) were produced as a basis for the exposure assessments of the Western Baltic Sea fish species. All exposure maps are available at https://github.com/GuiSPinto/cva_baltADAPT.

#### Species distribution maps

We developed species distribution maps for comparison with exposure factor maps as a basis for the exposure scoring. We acquired data from ICES BITS and BIAS (Baltic International Acoustic Survey) as well as the Baltic Acoustic Sprat Survey (BASS), all accessible via DATRAS (https://datras.ices.dk). For our study, we included catch per unit effort (CPUE) data from 2010 to 2019 for BITS and BIAS, and from 2015 to 2019 for BASS. Inverse distance weighting (IDW) was applied for interpolating species distributions according to a grid of 0.05° longitude × 0.05° latitude (~ 3 × 6 km^2^). Each cell reports the median of annual values recorded across the entire period for that specific cell. Species distribution maps are available at https://github.com/GuiSPinto/cva_baltADAPT.

### Expert scoring

Sensitivity scoring was conducted by a sub-group of the co-authors (20) representing expert knowledge with different educational/scientific backgrounds (aquatic ecology, fish and fisheries biology/ecology/oceanography). The heterogeneity of the group was assumed to cover expertise for the 22 different species selected. A sub-group of six co-authors familiar with Baltic Sea physical oceanography conducted the exposure scoring. A single questionnaire was prepared for the sensitivity assessment and exposure assessments were conducted in three separate questionnaires according to the three projection scenarios used (RCP 4.5 MOC and EOC, RCP 8.5 EOC).

We used a tally system for both expert sensitivity and exposure scorings^9^. Each expert distributed five tallies among the four different sensitivity/exposure categories (low, moderate, high, and very high) allowing the participants to express their expert certainty. Additionally, experts placed four tallies in three bin categories (negative, neutral and positive) to assess *Directional Effects* of climate-related environmental changes on the fish community during the sensitivity assessment. Eventually, experts also assessed *Data Quality* for both sensitivity and exposure assessments using scores from 0 to 3 with 3 = adequate data, 2 = limited data, 1 = scoring based on expert judgement only, 0 = no data available^9^. Results of the scoring exercises were exported from the online platform into the free software environment for statistical computing and graphics R^22^ for analysis.

### Data analysis

#### Sensitivity and exposure scores

For each species i, we calculated a weighted mean of the individual expert scores on each sensitivity attribute (SA) and exposure factor (EF)^9^:

2 $${X}_{i}=\frac{\left(\left(L\times 1\right)+\left(M\times 2\right)+\left(H\times 3\right)+\left(VH\times 4\right)\right)}{\left(L+M+H+VH\right)}$$

where X_i_ indicates either SA_i_ or EF_i_; L, M, H and VH are the number of tallies in the “low”, “moderate”, “high” and “very high” scoring bins, respectively.

Based on individual SA_i_ scores, we calculated sensitivity scores for each species by numerical averaging and additionally by using a logic rule^9^. According to the latter, species are scored into the VH category when ≥ 3 individual attributes have mean values ≥ 3.5, into the H category when ≥ 2 of the attributes have mean values ≥ 3.0, into the M category when ≥ 2 attributes have mean values ≥ 2.5, and into the L category when < 2 attributes have mean values ≥ 2.5. Usually a logic rule is preferred over numeric averages because attributes and factors are not intended to be correlated^29^. However, using numeric values allowed us to analyse the variability of SA_i_ between species using Principal Component Analysis (PCA) and Hierarchical Clustering (see below).

Since we only used temperature as an EF_i_ we could not apply the original logic rule^9^. We hence distributed scores into categories based on their mean numeric values (VH ≥ 3.25, H = 2.50–3.25, M = 1.75–2.49, L = 1.00–1.74).

#### Directional effects

We computed directional effects (DE) as a weighted mean of the individual expert scorings^9^:

3 $$\frac{{DE}_{i}=\left(\left(Pos\times 1\right)+\left(Neu\times 0\right)+\left(Neg\times -1\right)\right)}{P+Neu+Neg}$$

where *Pos* are the tallies scored in the “positive category”, *Neu* are the tallies scored in the “neutral category”, and *Neg* are the tallies scored in the “negative category”. We classified an overall negative effect when the score was < − 0.333, a neutral effect between − 0.333–0.333 and a positive effect when the score was > 0.333.

#### Vulnerability

We calculated the overall vulnerability of each species by multiplying the mean sensitivity and exposure scores. We distributed scores into vulnerability categories based on their mean numeric values (VH ≥ 3.25, H = 2.50–3.25, M = 1.75–2.49, L = 1.00–1.74).

#### Certainty analysis

We explored the variability of the assessments between experts based on a bootstrap analysis^9^. Scores were resampled 10^3^ times with replacement from the sensitivity and exposure assessments, and the process was repeated for each sensitivity and exposure category. Subsequently, we combined each run to calculate the final vulnerability score. The final certainty score represents the highest percentage of the computed vulnerability score distribution. We used the same approach to evaluate certainty for the direction of effects of climate on each fish species.

#### Software tool and libraries

All analyses and visualizations were performed in the free software environment for statistical computing and graphics R^22^ using Rstudio^23^ and the packages “tidyverse”^24^, “factoextra”^30^, “cluster”^31^ and “pheatmap”^32^. Digital topography data for the map in Fig. 1 were derived from “https://www.io-warnemuende.de/topografie-der-ostsee.html”.

## Results

### Sensitivity

#### Thermal tolerance of species and scoring of individual attributes

**Figure 3:**
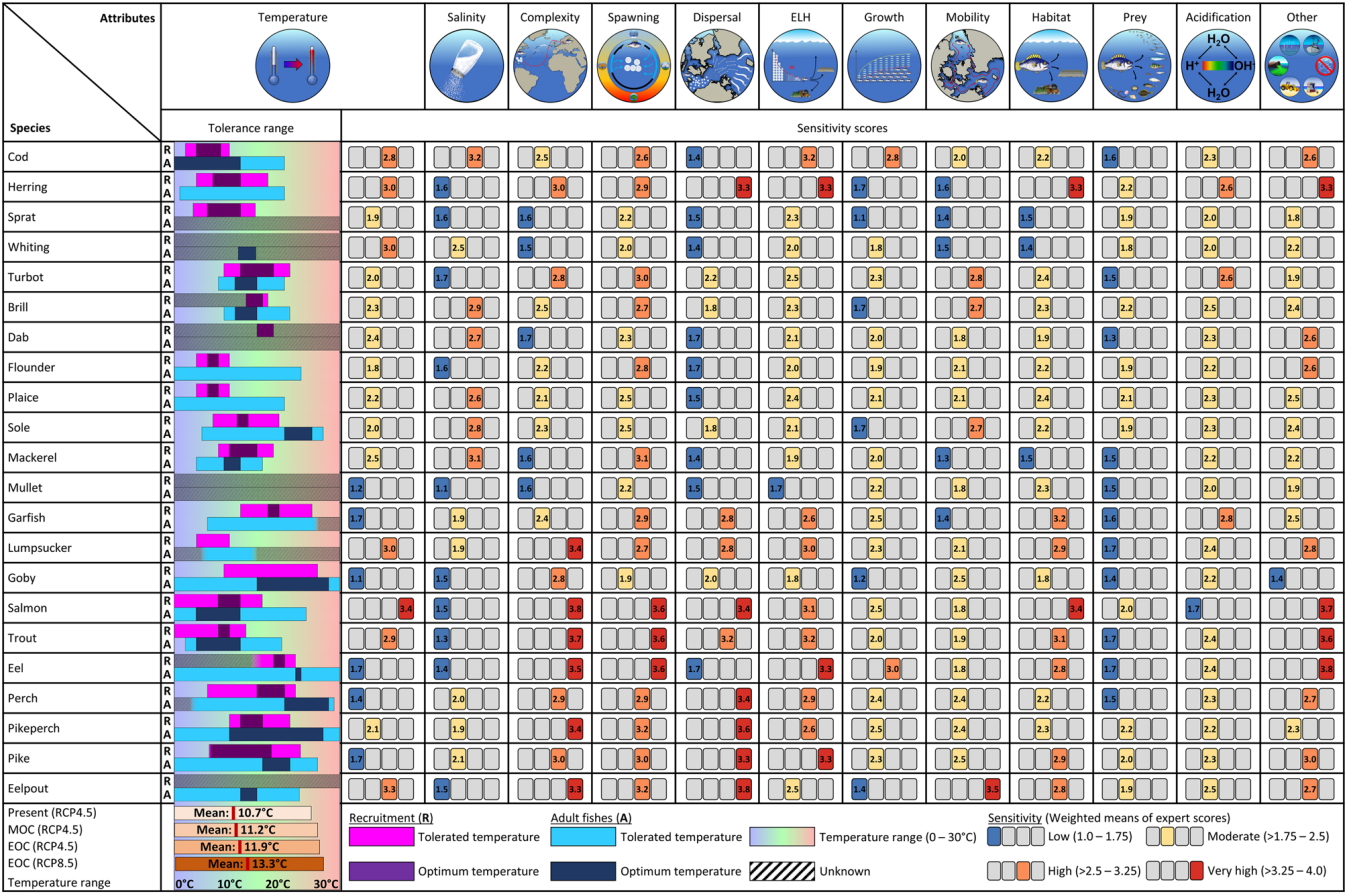
Summary of thermal tolerances and attribute-based sensitivity scores of Western Baltic fish species. Species names are according to Table 1; thermal tolerance ranges reflect the needs of species for recruitment (R) and adult stages (A) as found in the literature (see species profiles); sensitivity scorings represent the means calculated over the values provided by all experts; RCP 4.5 and 8.5 represent emission scenarios (see “Materials and methods”); *MOC* middle of the century, *EOC* end of centrury.

**Figure 4:**
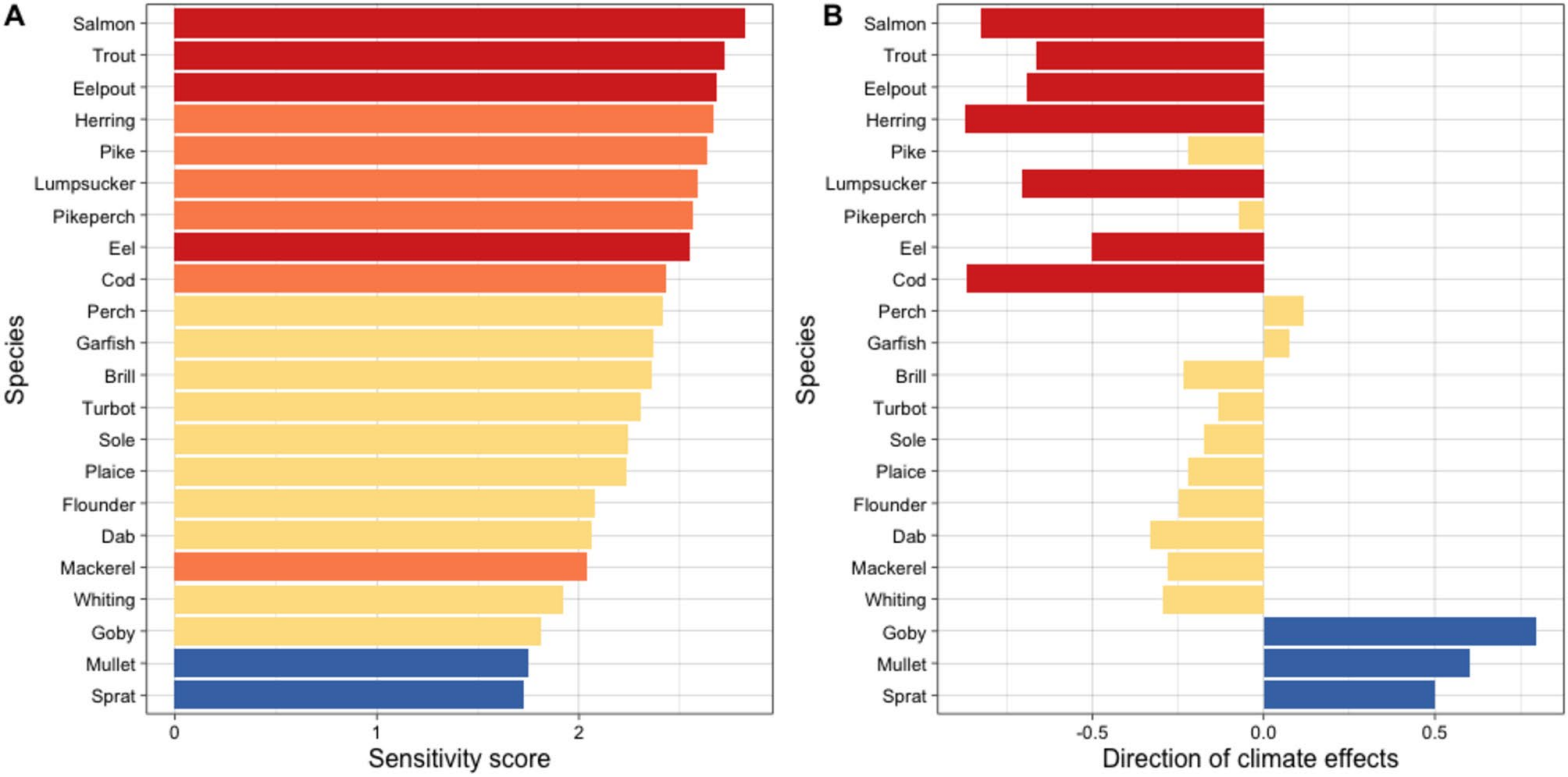
Sensitivity of Western Baltic fish species to climate change. (**A**) Species’ sensitivity calculated as the weighted mean over 12 individual sensitivity attributes and using all expert scores; colours represent sensitivity scores according to the logic rule: red—very high, orange—high, yellow—moderate, blue—low. (**B**) Directional effects of climate change obtained as weighted means over all expert scores; colours represent categorization according to the logic rule: red—negative, yellow—neutral, blue—positive.

**Figure 5:**
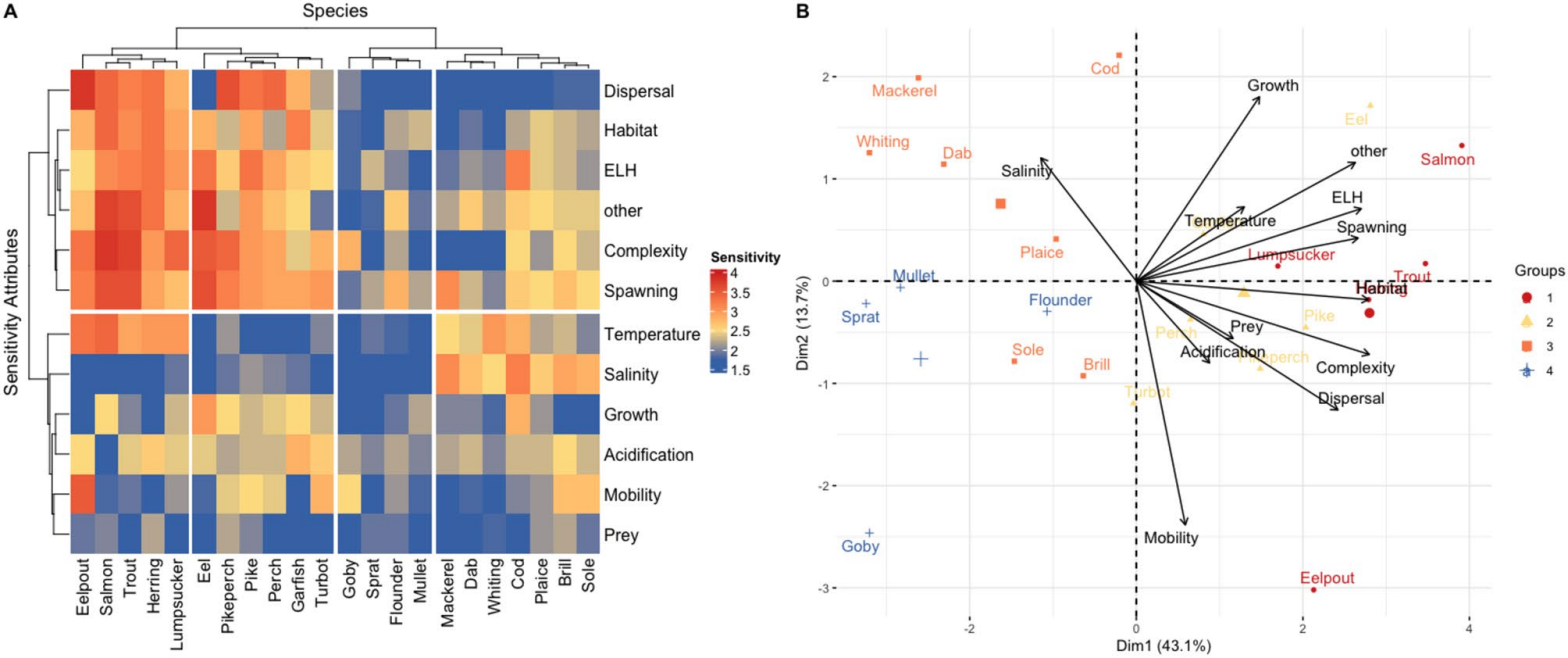
Relationships between sensitivity attributes and Western Baltic fish species. (**A**) Heatmap showing sensitivity attributes (rows) and species (columns); dendrograms and map splitting reflect the hierarchical clustering. (**B**) Principal Component Analysis biplot projecting attributes and species on the first two dimensions (Dim1, Dim2); colours of species groups assigned according to hierarchical clustering; complete attribute names and descriptions are provided in Table 2; species overplotted by “Habitat” is “Herring”.

Figure 3 provides an overview of thermal tolerances and mean (across all experts) scores for the 12 attributes quantifying the sensitivity to climate change of 22 fish species inhabiting the Western Baltic Sea. Thermal tolerances are indicated because of our focus on exposure to temperature changes. Scores of individual climate sensitivity attributes provide the basis for the evaluation of overall species’ sensitivity (Figs. 4 and 5).

#### Sensitivity scores and directional effects

Mean numeric sensitivity scores derived from our expert assessment largely match the categorization according to the logic rule (Fig. 4A). 10 out of 22 species were assessed to have a high or very high sensitivity to climate change. In addition to salmon, trout and eelpout, the sensitivity of eel to climate change was rated as very high while the numeric score is a little lower compared to the other three species. Other species considered to be of high sensitivity were two traditional fishing targets as cod and herring, pike and pikeperch (freshwater origin), and lumpsucker. Additionally, the seasonally immigrating mackerel was rated to have a high sensitivity while the numeric score was comparatively low. The high sensitivity of mackerel resulted from high scores for the three attributes *Spawning* (full names of the attributes given in Table 2), *Temperature* and *Salinity*, while other attributes were assessed to be relatively low (Fig. 5). On the other end, experts scored mullet and sprat to be least sensitive. Overall, 10 out of 22 species were assessed to have a moderate sensitivity to climate change, including all flatfish species.

Numeric scores for the directional effects of climate change matched the categorization according to the logic rule and all neutral scores were significantly higher than the negative and lower than the positive scores (Fig. 4B). Furthermore, directional effects largely reflect the assessment of overall fish sensitivity. Very highly and highly sensitive species were generally assessed to be negatively affected by climate change with the exception of pike and pikeperch, which were categorized as neutral (Fig. 4A). Cod and herring, the traditionally most important fisheries targets, were rated to likely experience the strongest negative effects due to climate change. On the other end of the spectrum, species with the lowest sensitivity as mullet and sprat were assessed to profit from climate change (positive effect). The highest positive effect of climate change is indicated for the invasive goby that was rated with being only moderately sensitive, but with a very low numeric score.

#### Importance of sensitivity attributes

We analysed the importance of individual attributes for the sensitivity of fish species to climate change using Hierarchical Clustering (HC) and Principal Component Analysis (PCA). According to the expert assessment, life-cycle attributes mainly determine the sensitivity of species to climate change. A main “attribute” cluster (upper left part of the heatmap in Fig. 5A) groups together species displaying complex reproductive strategies (*Complexity*), including temporally-restricted and few spawing events (*Spawning*) and low dispersal rates of early life stages (*Dispersal*), which additionally have specific survival and settlement requirements (*ELH*). Further attributes of species lumped together in this cluster are specific habitat requirements (*Habitat*) and additional stress factors (*Other*). Typically, these attributes are distinctive of fish that have a high or very high sensitivity to climate change. Main representatives of these sensitive groups (denoted by 1 and 2 in Fig. 5B) include (a) salmon, trout and eel, which have diadromous life-cycles; (b) herring (specific habitats are required for their benthic eggs); (c) eelpout and lumpsucker as they do depend on a form of brood care; and (d) pike and pikeperch, which have freshwater origin and in the Western Baltic Sea live at the limits of their distribution. Species of group 1 differ from those of group 2 mainly because of the sentivitity to temperature, which characterizes the second cluster (Fig. 5B).

The relationship between complex life-cyles and the sensitivity to climate change is also demonstrated by the results of the PCA (Fig. 5B). Species considered to be of high and very high sensitivity (groups 1 and 2) are positively related to the dominant mode of variability (Dim1) as are the traits from the main “sensitivity attribute” cluster (upper cluster in Fig. 5A). Species of low to moderate sensitivity (groups 3 and 4) are consequently negatively related to Dim1. These groups contain almost exclusively marine species (and goby), including almost all flatfish species (except turbot) that generally have less challenging life-cycles (Fig. 5A). A sub-cluster (group 3, Fig. 5B) is positively related to the second mode of variability (Dim2) and hence characterized by high sensitivity to salinity. Species of this groups include mackerel (pelagic), two bottom-dwelling flatfish as dab and plaice, and the demersal gadids cod and whiting. Negatively related to Dim2 are species considered to be of low mobility such as the highly-sensitive eelpout and the low-sensitive goby.

Experts additionally rated the quality of the data available for assessing the sensitivity of the species (https://github.com/GuiSPinto/cva_baltADAPT). The two commercially-important species cod and herring are considered as those best studied. A general lack of information on the attribute *Acidification*. Data availability on some sensitivity attributes (*Other*, ELH, *Temperature*, *Complexity*) was limited for some species.

### Exposure to temperature change

**Figure 6:**
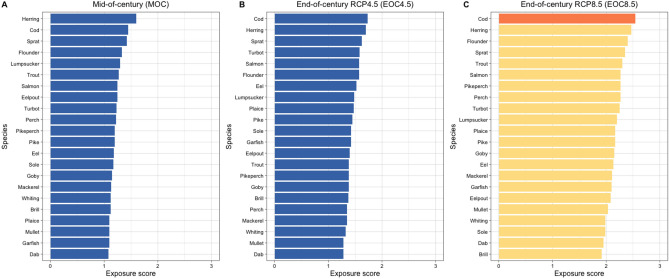
Exposure of the Western Baltic fish species to climate change. Exposure scores for (**A**) RCP 4.5 mid-of-century climate scenario, (**B**) RCP 4.5 end-of-century climate scenario, and (**C**) RCP 8.5 end-of-century climate scenario. Colours represent sensitivity scores according to the logic rule: orange—high, yellow—moderate, blue—low.

Exposure to climate-related warming was rated based on the overlap of individual spatial distributions of fish species with temperature projections according to “mid of century” (MOC; only RCP 4.5) and “end of century” (EOC; RCP 4.5 and 8.5) scenarios (Fig. 6). Similar to the sensitivity, numeric scores of exposure largely match the categorization obtained using our modified logic rule. Exposure to temperature under both RCP 4.5 (MOC and EOC) scenarios was generally rated to be low, with a slight increase in the mean numeric score towards later in the century (1.2 and 1.5 for MOC and EOC, respectively). Exposure scores for EOC increase under RCP 8.5 to largely moderate (mean score of 2.2), except for cod that is rated as highly exposed (mean score of 2.9). The commercially important species cod, herring and sprat have generally the highest exposure values. Species such as dab and mullet are often found at the lower exposure range. Data quality for the exposure ratings was high for the traditional target species of the fishery but low for less abundant marine, freshwater-marine, and freshwater-brackish species (Table 1; https://github.com/GuiSPinto/cva_baltADAPT).

### Vulnerability

**Figure 7:**
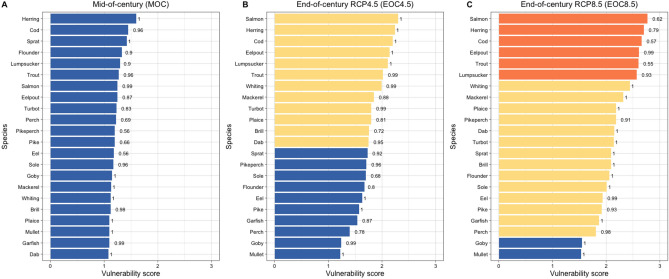
Vulnerability of Western Baltic fish species to climate change. Vunerability scores are reporterd for (A) RCP 4.5 mid-of-century climate scenario, (**B**) RCP 4.5 end-of-century climate scenario, and (**C**) RCP 8.5 end-of-century climate scenario. Colours describe the intensity of sensitivity scores according to the logic rule: orange—high, yellow—moderate, blue—low. Certainty estimates derived by bootstrapping are added as numbers next to individual bars.

Combing scores on overall sensitivity for each of the 22 fish species with those on the exposure to future warming yielded in the final assessment of their vulnerability to climate change (Fig. 7). Numeric scores (mean = 1.6) as well as the categorization according to the logic rule indicate a low vulnerability of all species until the mid of the century (Fig. 7A). Vulnerability increases towards the end of the century and varies according to emission scenario. Under RCP 4.5, the overall numeric score only slightly increased compared to the earlier period (mean = 1.8) but the categorization according to the logic rule indicates a split of the fish community in species with low and moderate vulnerability (Fig. 7B). Under the high emission scenario (RCP 8.5; mean = 2.2), most of the fish species are rated to be moderately vulnerable and only goby and mullet stay with low vulnerability (Fig. 7C). The commercially important cod and herring, the diadromous fish as salmon and trout, eelpout, and lumpsucker all exhibit a high vulnerability to climate change.

We also investigated the certainty of vulnerability scores using bootstrapping and found high values close to 1 (Fig. 7). Notable exceptions are the mostly freshwater species perch, pike and pikeperch as well as a catadromous species as eel under the MOC scenario, and some species under the RCP 8.5 scenario at the end of the century (i.e., salmon, cod, and trout).

## Discussion

We here report on a first climate vulnerability assessment (CVA) of a fish community in the Baltic Sea where knowledge on how climate change will likely affect entire fish communities is deficient. A general lack of quantitative data for many of the fish species is also characteristic for our study area, the Western Baltic Sea, and we hence implemented a qualitative, expert-based approach that has been applied to many areas of the world ocean and returned promising results^4,6–9^. Because of the uniqueness of the Western Baltic Sea ecosystem, a transition basin between the more saline areas bordering the North Sea (i.e., Skagerrak and Kattegat) and the low saline Baltic proper^33^, we had to adjust the attribute and category descriptions for the sensitivity assessment to the special conditions of our study region. We supported the assessment of the 12 species attributes by comprehensive fish profiles that were the result of an extensive review of the available literature. The literature is unsurprisingly dominated by studies on cod and herring, the two traditional commercial targets, an aspect that may have contributed to the dominant perception of their high sensitivity to climate change. Data quality assessments especially also revealed a lack of information on the potential effects on single attributes, and especially on ocean acidification as an important climate change effect for almost all species (but see^34^). To provide a reliable CVA, we made an effort to assemble a group of participating scientists that represented broad ecological expertise on the 22 fish species considered. Nevertheless, a broader and more international participation of experts may contribute to sharpening potential future CVA outcomes.

A major result of our study is that marine and freshwater species with complex life-cycles attributes were assessed to have high and very high sensitivity to climate change effects. Highly sensitive species are especially anadromous salmonids (salmon and trout), and the catadromous eel. Such diadromous life-cycles are characterized by complex spawning migrations between fresh- and seawater habitats, with distinct spawning periods and specific requirements for the well-being and survival of their early life stages^35–37^. The typically long migrations of diadromous species expose them to a multitude of anthropogenic and natural pressures limiting e.g. connectivity between habitats^38–40^. The state of European eel populations showcases their high sensitivity, not only to climate change, but to a multitude of cumulative pressures. European eel is currently assessed as critically endangered with a dramatic decline in recruitment and no signs of recovery^41^.

Additionally to diadromous species, fish displaying further aspects characteristic of complex life-history strategies have been rated highly sensitive by experts. This group includes species typically requiring specialised habitats for benthic egg deposition such as in herring and garfish^42,43^, and species conducting a form of brood care like eelpout and lumpsucker^44,45^. Also species of freshwater origin that live in the Western Baltic Sea at the limits of their salinity tolerance (i.e., pike, perch, and pikeperch) are considered highly sensitive. These latter species display complex reproductive strategies, require special vegetation for spawning^46^, and have a pronounced homing behavior^47–50^.

In contrast to species with complex life-history stategies, successful invaders to the Baltic Sea such as goby, mullet and sprat were assessed to be least sensitive and may hence profit from climate change. For goby, low sensitivity scores are in accordance with the literature that reports this species to benefit from climate-related environmental changes^51^, because of a broad thermal tolerance^50,52^ and a high potential for adaptation to local environmental conditions^53^. In contrast to the invading goby, the two least sensitive species in our assessment, i.e. mullet and sprat, reached the Baltic Sea by range expansions and both species adapted to the wide range of salinities and temperatures. Sprat is widely established and occupies a broad range of the basin, from southern to northern areas^54^, while a regular occurrence of mullet in the north of the Baltic Sea is a recent phenomenon^55^.

A further obvious result of our study is that experts assessed the traditionally dominating fishing targets in the Western Baltic, cod and herring, as highly sensitive to climate change. In contrast to other highly sensitive species, the sensitivity of cod is not related to any complex life-cycle attributes, but to its dependence on higher salinities^56^. Generally, cod is considered to suffer from the effects of climate change in the Western Baltic Sea, especially warming, while the exact process is still unclear^18,57–59^. In contrast, herring is tolerant to a wide range of salinity changes^60,61^, but belongs to the group of climate sensitive species because of its rather complex life cycle. Demersal spawning of herring requires specific habitats such as vegetated coastal waters, i.e., estuaries, lagoons, and bays^62^. Egg development and recruitment are therefore highly dependent on environmental conditions and especially the temperature regime in the littoral zone. Our exposure analysis revealed that essential inshore nursery habitats will face drastic drastic future warming, potentially limiting recruitment and reducing stock productivity. Furthermore, milder winter temperatures in recent years have likely caused a shift in spawning phenology, resulting in a potential mismatch of prey items for herring larvae during their critical first-feeding stage^19^.

In contrast to cod and herring, our expert-based assessement revealed flatfish species to be less sensitive to the effects of climate change in the Western Baltic Sea. The recent drastic increase in plaice in the area may be a result of their low climate sensitivity^63^. The increase in the bottom living plaice stock is surprising given the increasing extension of hypoxic bottom zones in the Western Baltic Sea^12–14^. Hypoxic conditions are expected to decrease the availability of suitable habitats for flatfish, but also cod. However, physiological studies point towards a stronger resilience of flatfish compares to the combined effects of warming and deoxygenation compared to cod^64^.

Experts considered exposure of Western Baltic fish species to warming to be mainly low under the moderate RCP 4.5 scenario, increasing to largely moderate only under the most severe emission scneario RCP 8.5. The overall low to moderate exposure mainly reflect that temperature projections indicate a weaker warming for the Western Baltic Sea compared to the eastern and northern Baltic Sea areas^10^. As a result most of the area considered in our study fell within the low or moderate warming category. A further reason for the low exposure is that we considered the period of 2010–2019 as the baseline for the categorization of warming. This decade already experienced a significant degree of warming compared to pre-industrial times thus reducing the chances of detecting major significant differences when contrasted with future projections^10^. Our exposure rating hence quantifies the extent of additional future warming in the Western Baltic Sea.

The exposure assessment generally bears most of the room for improvement in a future CVA for the Western Baltic fish community. We used the best available survey data to assess the spatial overlap of the species with future thermal conditions, however these surveys do not sufficiently cover the distribution ranges of all species as especially known for cod^57,65^. Moreover, the data cover only some seasons, not accounting for seasonal migrations. Additionally, many of the species that are traditionally of no high commercial importance such as pike, perch and pikeperch are not targeted by the existing monitoring programmes, hence no numeric information on their distribution exists.

A further limitation of our assessment is that we only focused on the exposure to warming because of uncertainties in the future projections of salinity and oxygen conditions^10,21,27^. Furthermore, detrimental oxygen conditions are assumed to be mainly determined by eutrophication, while the importance of ocean warming for the increased occurrence of oxygen minimum zones needs further investigations^12,66^. Variability in salinity is also crucially important for fish species in the Western Baltic Sea, an area with a dynamic hydrography^15^, since they will likely affect the productivity and distribution of all the species (documented for each species in the species profiles; see “Materials and methods”). Future exposure ratings need to better include these in the CVA when more reliable projections of salinity and oxygen are available. Future assessments may additionally comprise indices for future changes in plankton productivity and ocean acidification^9^.

Combing sensitivity and exposure provided vulnerability assessments of the Western Baltic fish species to climate change. Vulnerability is expected to be generally low until the middle of the century, and increased to moderately or high depending of emission scenario and species considered. Vulnerability assessments largely reflect the sensitivity of the species, because exposure was generally less variable. The most vulnerable species include the traditional fishing targets cod and herring as well as the salmonids salmon and trout, which altogether are also found under the species with highest sensitivity and exposure scores. Highly vulnerable outcomes are also found for lumpsucker and eelpout, two species with a high sensitivity but comparatively lower exposure scores. Other highly sensitive species like the mainly freshwater species (i.e. pike, perch, and pikeperch) are categorized as moderately vulnerable due to their lower exposure ratings, a result that holds true also for eel. Lowest vulnerability is found for goby and mullet, mainly because of their low sensivitity scores.

## Conclusions

Our study revealed a dichotomy in climate change vulnerability within the fish community of the Western Baltic Sea. Traditional fishing targets and species with complex life histories face heightened risks, whereas invasive or adaptable species might thrive under changing conditions. Such a scenario presents significant ecological and economic implications, particularly for local fisheries traditionally reliant on sensitive species like cod and herring. Our findings furthermore demonstrate the complex interplay between life-history traits and climate change vulnerability in marine fish communities. Our analysis, while methodologically robust, also highlights the limitations inherent in such assessments. The dearth of comprehensive ecological data, particularly on non-commercial species, and the focus on temperature as a primary climate factor without fully integrating other critical parameters like salinity and oxygen levels, suggest areas for improvement in future studies. Overall, our study is a call for the urgent development of tailored adaptation efforts addressing existing but especially future effects of climate change on fish and fisheries in the Western Baltic Sea to navigate this endangered fisheries systems into a sustainable future.

## Data Availability

All data are available from the corresponding author on request.
