# Correction: A climate vulnerability assessment of the fish community in the Western Baltic Sea

**DOI:** 10.1038/s41598-025-04750-6

**Published:** 2025-06-06

**Authors:** Dorothee Moll, Harald Asmus, Alexandra Blöcker, Uwe Böttcher, Jan Conradt, Leonie Färber, Nicole Funk, Steffen Funk, Helene Gutte, Hans-Harald Hinrichsen, Paul Kotterba, Uwe Krumme, Frane Madiraca, H. E. Markus Meier, Steffi Meyer, Timo Moritz, Saskia A. Otto, Guilherme Pinto, Patrick Polte, Marie-Catherine Riekhof, Victoria Sarrazin, Heike Schwermer, Marco Scotti, Rudi Voss, Helmut Winkler, Christian Möllmann

**Affiliations:** 1 Thuenen Institute of Baltic Sea Fisheries, Rostock, Germany; 2 Alfred-Wegener Institute for Polar and Marine Research, Wadden Sea Station Sylt, List, Germany; 3 Institute of Marine Ecosystem and Fishery Science, Center for Earth System Research and Sustainability (CEN), Hamburg University, Hamburg, Germany; 4 Marine Ecology Research Division, GEOMAR Helmholtz Centre for Ocean Research Kiel, Kiel, Germany; 5 Department of Physical Oceanography and Instrumentation, Leibniz Institute for Baltic Sea Research Warnemünde, Rostock, Germany; 6 BioConsult GmbH & Co. KG, Bremen, Germany; 7 Stiftung Deutsches Meeresmuseum – Museum für Meereskunde und Fischerei, Deutsches Meeresmuseum, Stralsund, Germany; 8 German Centre for Integrative Biodiversity Research (iDiv), Halle-Jena-Leipzig, Leipzig, Germany; 9 Center for Ocean and Society (CeOS), Christian-Albrechts-University Kiel, Kiel, Germany; 10 Leibniz Institute for Biodiversity Change Analysis (LIB), Museum of Nature – Zoology, Hamburg, Germany; 11 Institute of Biosciences and Bioresources, National Research Council of Italy, Firenze, Italy; 12 Department of Zoology, University of Rostock, Rostock, Germany

Correction to: *Scientific Reports* 10.1038/s41598-024-67029-2, published online 13 July 2024

Heike Schwermer was omitted from the author list in the original version of this Article.

In addition, the Funding section was incomplete.

“Open Access funding enabled and organized by Projekt DEAL.”

now reads:

“Open Access funding enabled and organized by Projekt DEAL. We acknowledge financial support from the Open Access Publication Fund of Universität Hamburg.”

The original Article has been corrected.

